# Supplementary material for: Experimental determination of excitonic band structures of single-walled carbon nanotubes using circular dichroism spectra
Source: Nat Commun. 2016 Oct 5;7:12899. doi: 10.1038/ncomms12899 (PMC5113158; doi:10.1038/ncomms12899)
Supplement: Supplementary Information — Supplementary Figures 1-12, Supplementary Table 1, Supplementary Methods and Supplementary References [file ncomms12899-s1.pdf]

Supplementary Figure.

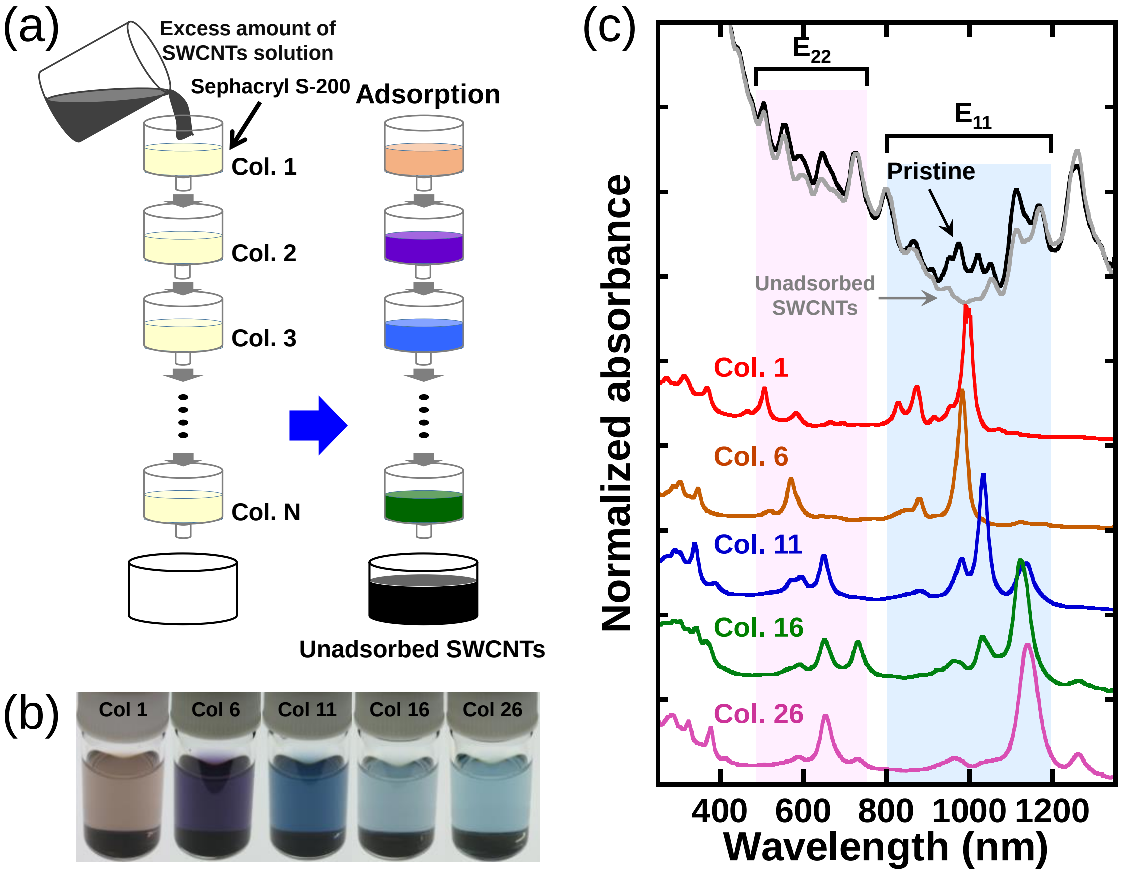

Supplementary Figure 1. The first separation utilizing overloading selective adsorption.

(a) Schematic diagram of overloading selective adsorption. (b) Photograph of the separated samples: Col.1, 6, 11, 16 and 26. (c) Optical absorption spectra of the samples. The spectra for pristine and unadsorbed SWCNTs are indicated by black and gray lines, respectively.

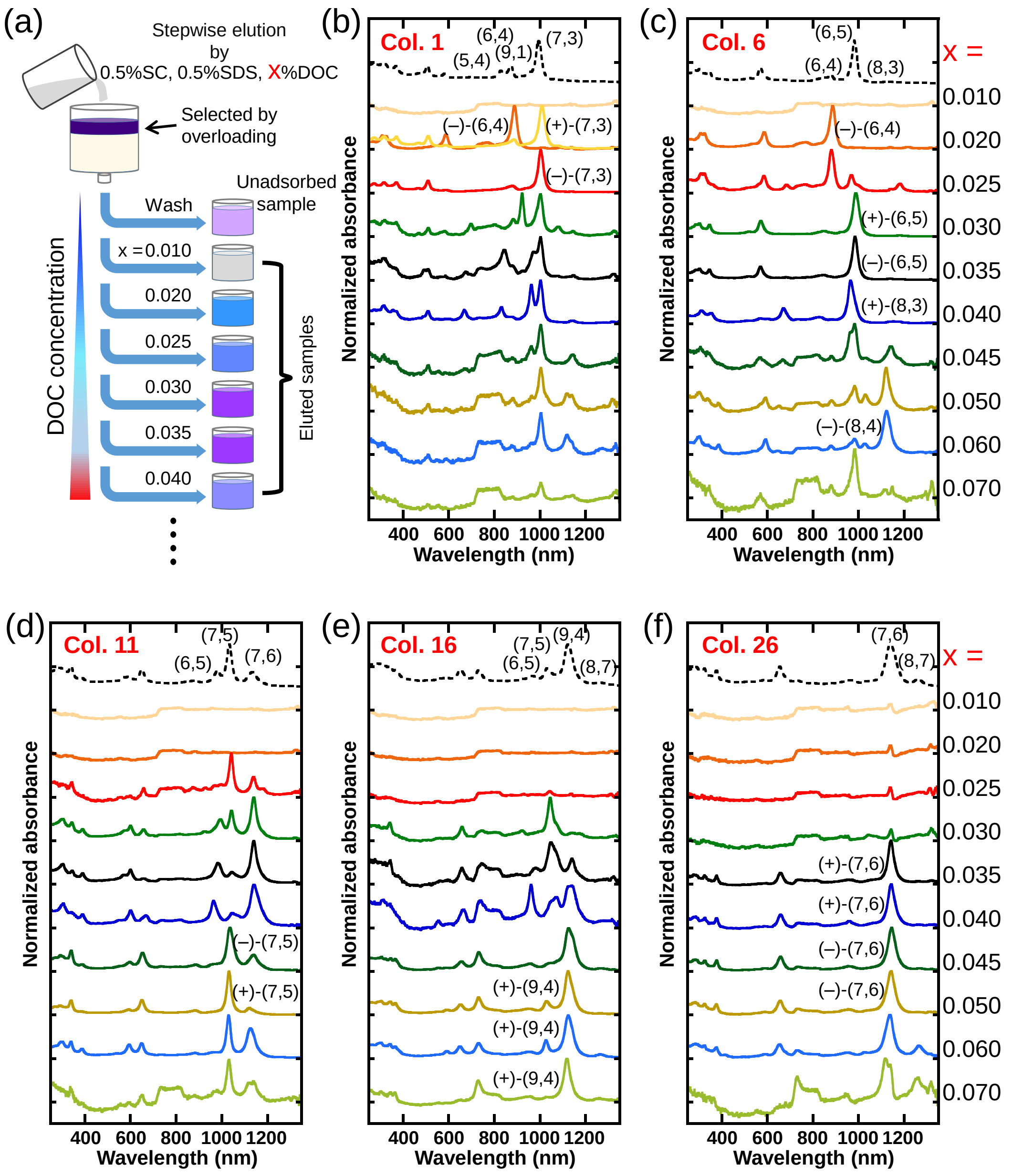

Supplementary Figure 2. The second separation utilizing stepwise elution.

(a) Schematic diagram of stepwise elution chromatography. (b-f) Optical absorption spectra of SWCNTs separated by stepwise elution using the samples obtained from the first separation: Col.1 (b), Col.6 (c), Col.11 (d), Col.16 (e), Col.26 (f). The surfactant concentrations used for the eluents were changed from 0.01 to 0.07% DOC in 0.5% SC and 0.5% SDS, as shown in Supplementary Figure 2. Note that in (c), (–)-(6,4) and (+)-(7,3) correspond to earlier and later eluted fractions, respectively. (–)- and (+)-(6,4) could be eluted at concentrations of 0.015% and 0.02% DOC in 0.5% SC and 0.5% SDS (data not shown).

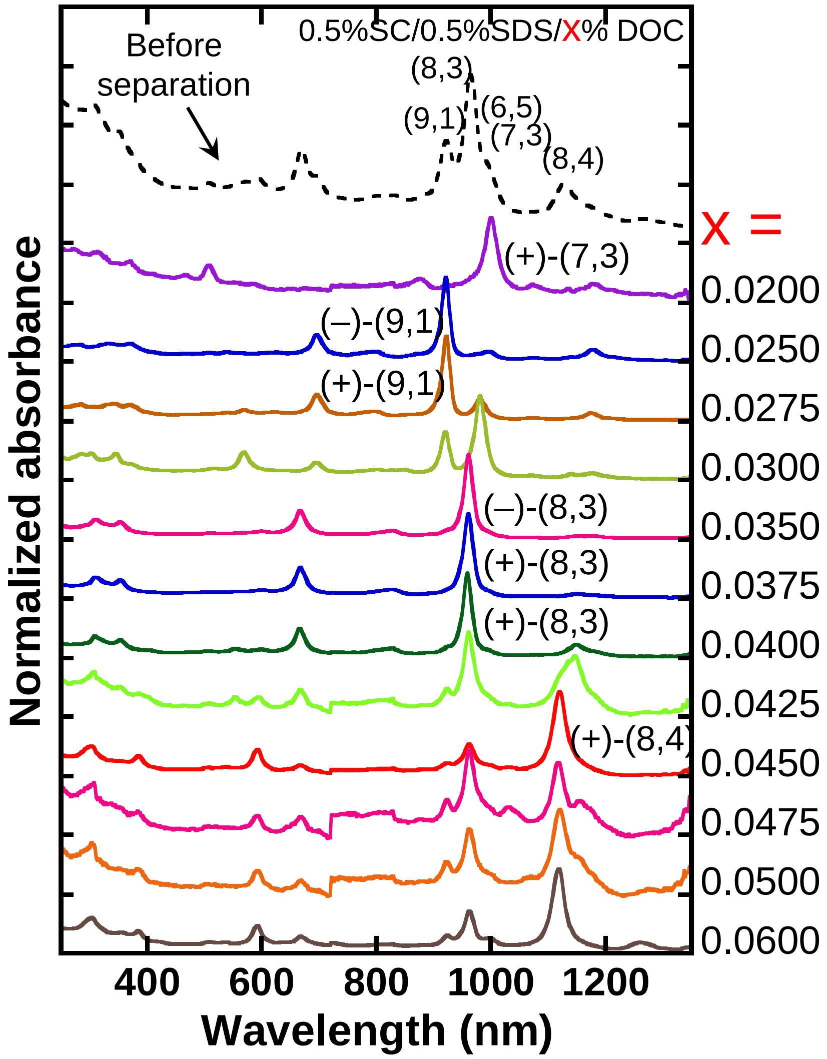

Supplementary Figure 3. Optical absorption spectra of separated SWCNTs using a SWCNT dispersion prepared under different conditions.

The surfactant concentrations used for the eluents were changed from 0.02 to 0.06% DOC in 0.5% SC and 0.5% SDS, as shown in Supplementary Figure 3.

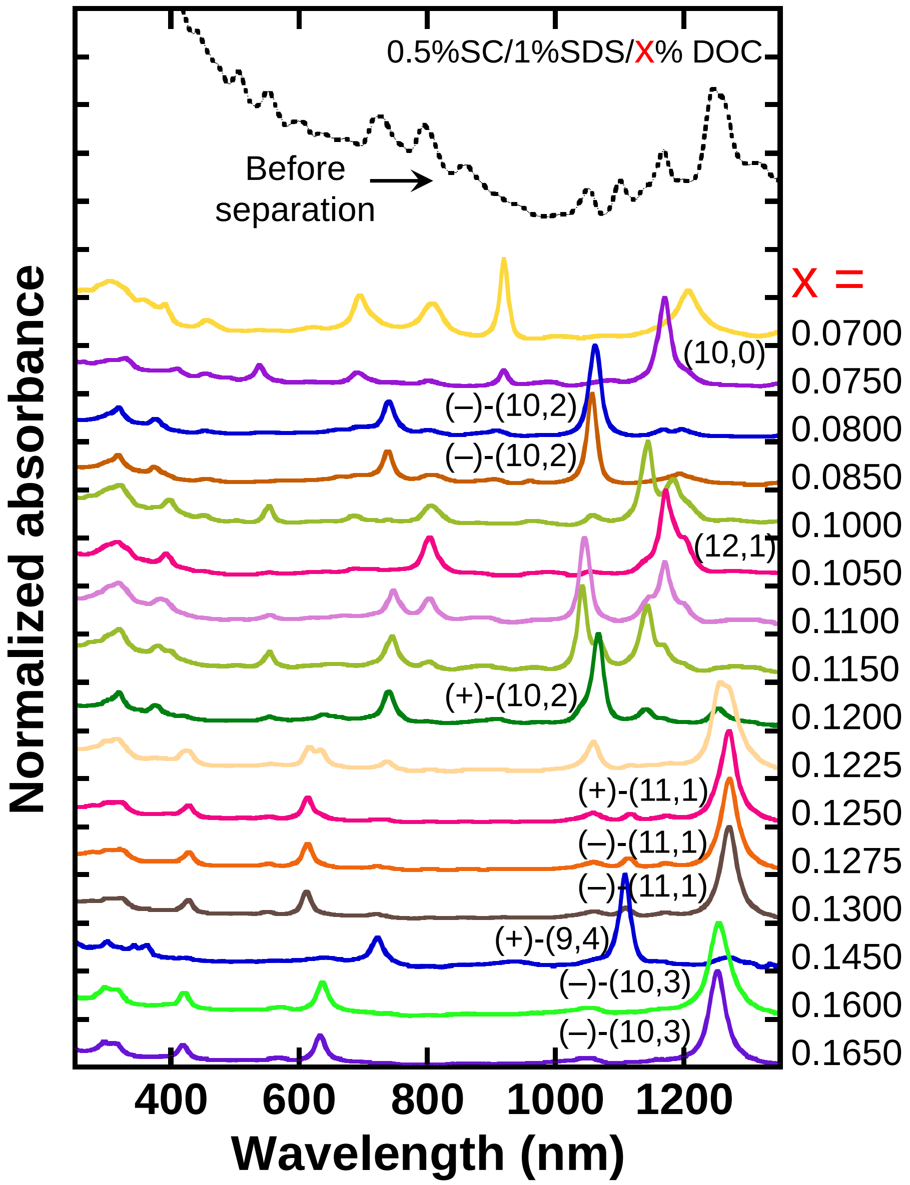

Supplementary Figure 4. Optical absorption spectra of separated SWCNTs with large diameters. The surfactant concentrations used for the eluents were changed from 0.07 to 0.165% DOC in 0.5% SC and 1.0% SDS, as shown in Supplementary Figure S4.

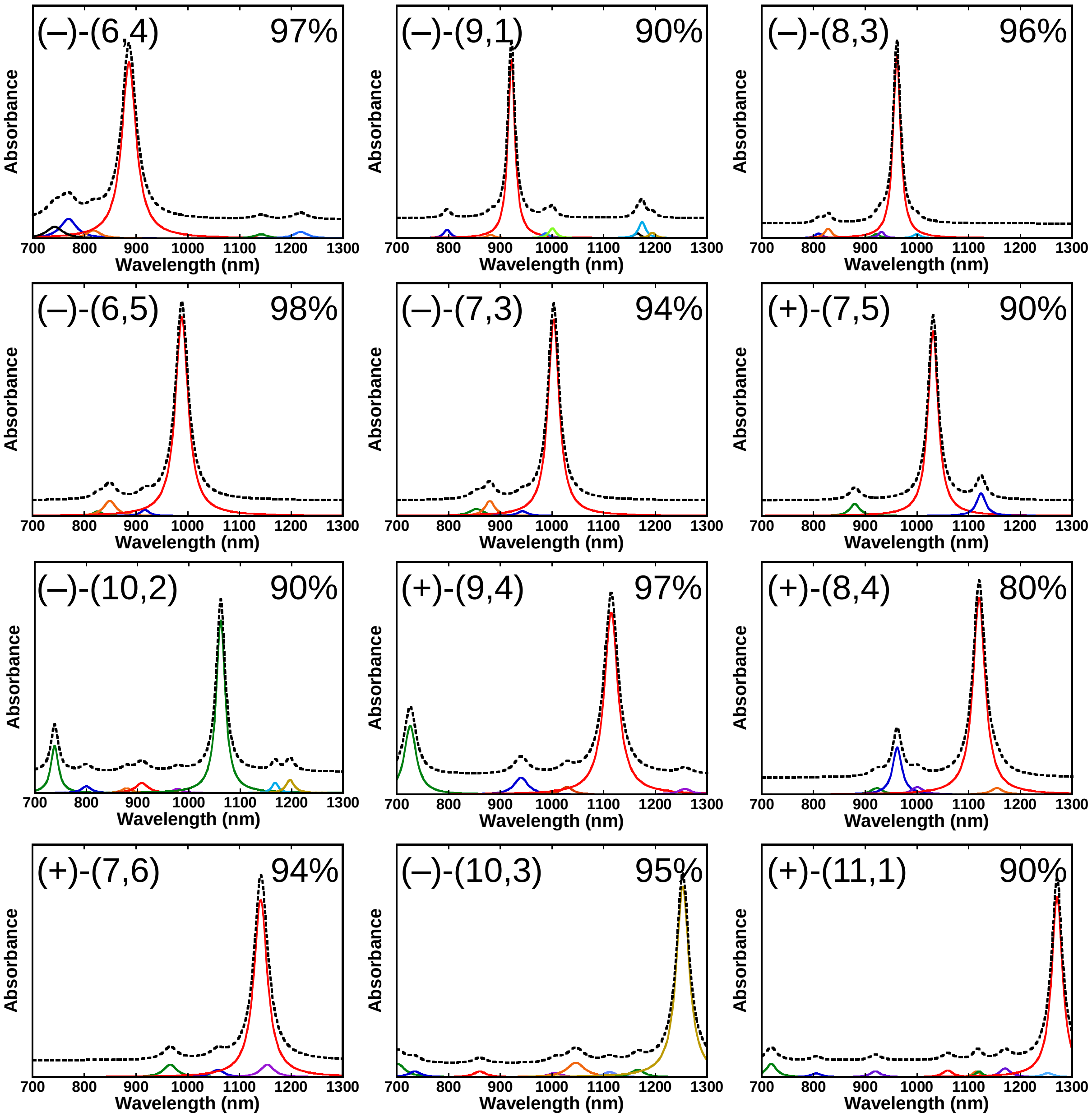

Supplementary Figure 5. Evaluation of the integrated absorption intensity ratio.

The integrated intensity ratio of the absorption of the major chirality peak to the sum of all chirality peaks was calculated for 12 (n,m) species using PeakFit software.

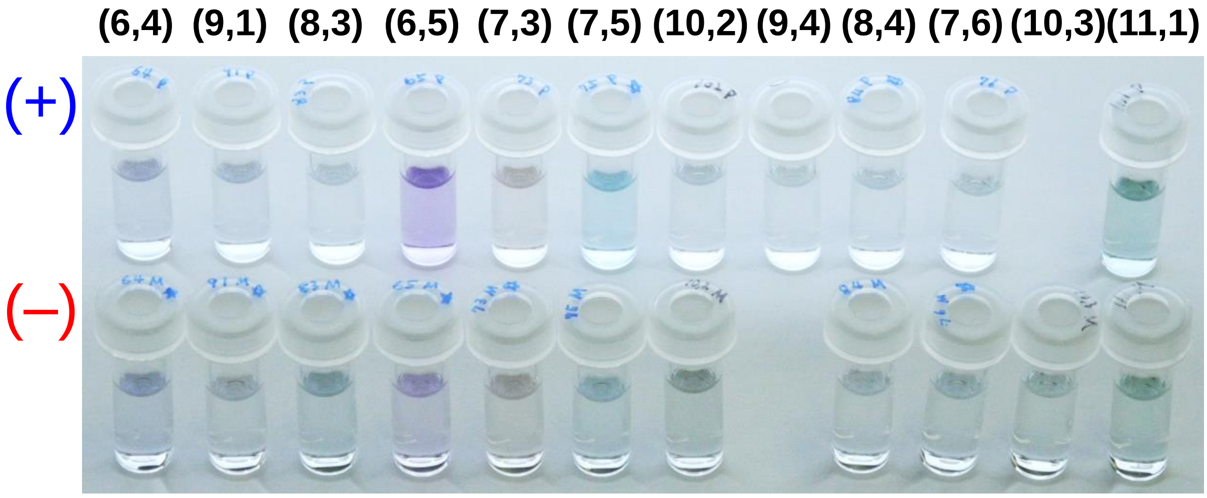

Supplementary Figure 6. Photograph of the sorted enantiomers of 12 single-chirality SWCNTs.

The upper and lower samples are (+)- and (–)-enantiomers of the (*n*,*m*) SWCNTs, respectively.

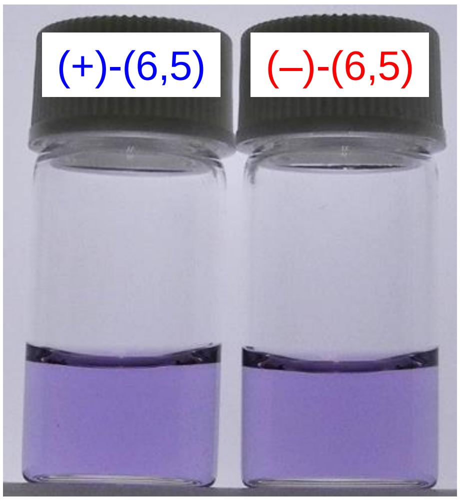

Supplementary Figure 7. Photograph of (+)- and (–)-(6,5) SWCNTs solutions.

The concentration of the solutions was adjusted based on the optical absorbance intensity.

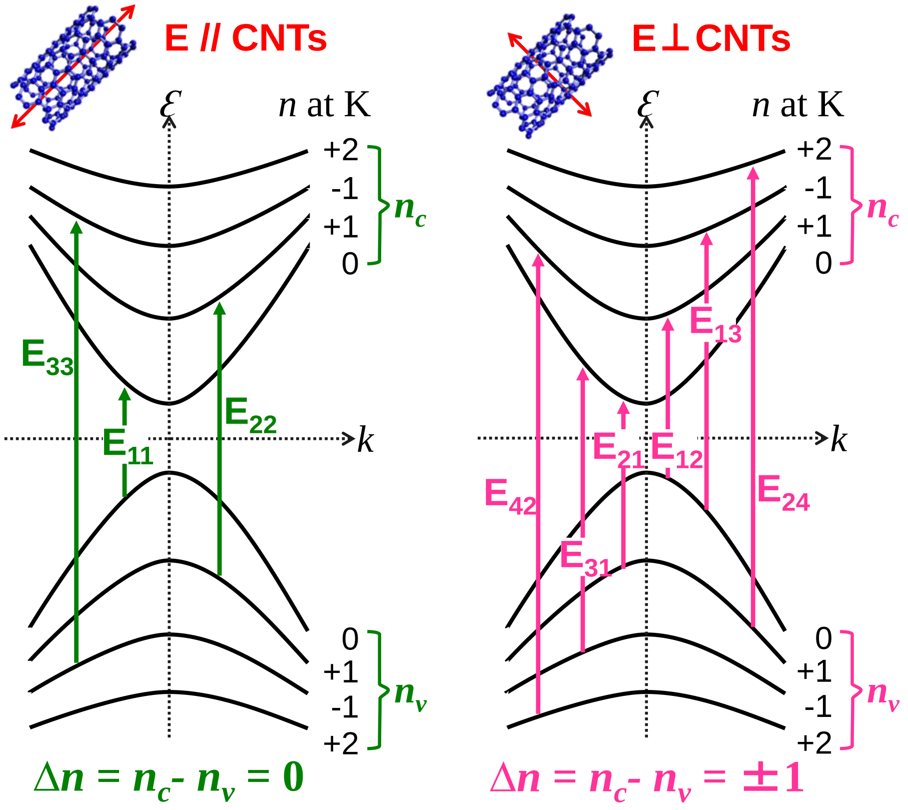

Supplementary Figure 8. Energy bands and allowed optical transitions of a semiconducting SWCNT. The band indices *n* (n_c and n_v) at the K point are labelled for a chiral vector. The allowed optical transitions are indicated by green and pink arrows for incident light polarized parallel (left) and perpendicular (right) to the nanotube axis, respectively. Allowed optical transitions between the valence band n_v and the conduction band n_c in a semiconducting SWCNT are determined by Δn, where $\Delta n = n_c - n_v$. The allowed transitions satisfy $\Delta n = 0$ or $\Delta n = \pm 1$ for incident light polarized parallel or perpendicular to the SWCNT axis, respectively[1,2].

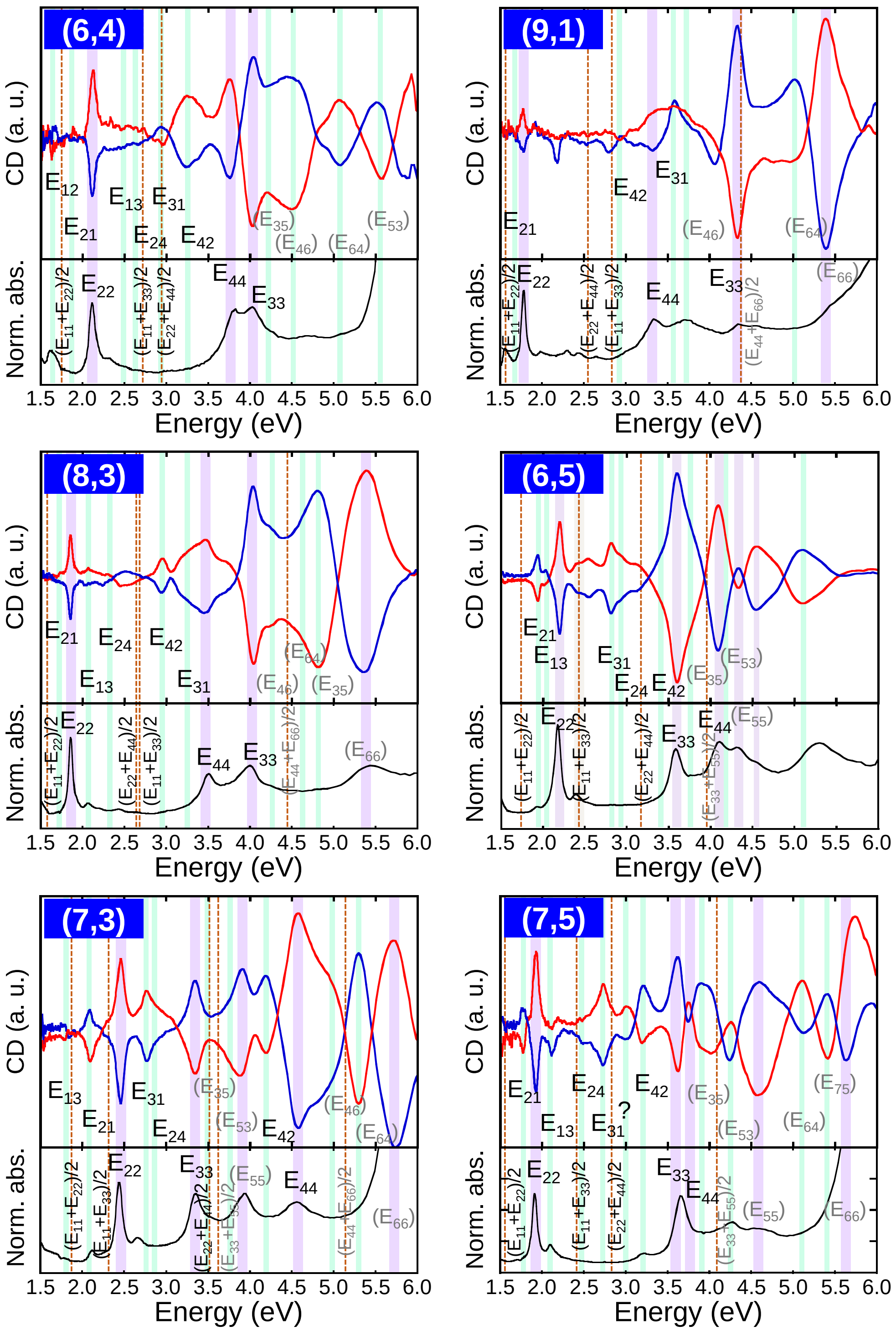

Supplementary Figure 9. Assignment results of observed optical transitions for 12 (*n*,*m*) SWCNTs. Vertical dashed lines correspond to the center positions of the pair of E_{ij} transitions. The optical transitions corresponding to Δn = 0 and Δn = ±1 are denoted as purple and green strips, respectively.

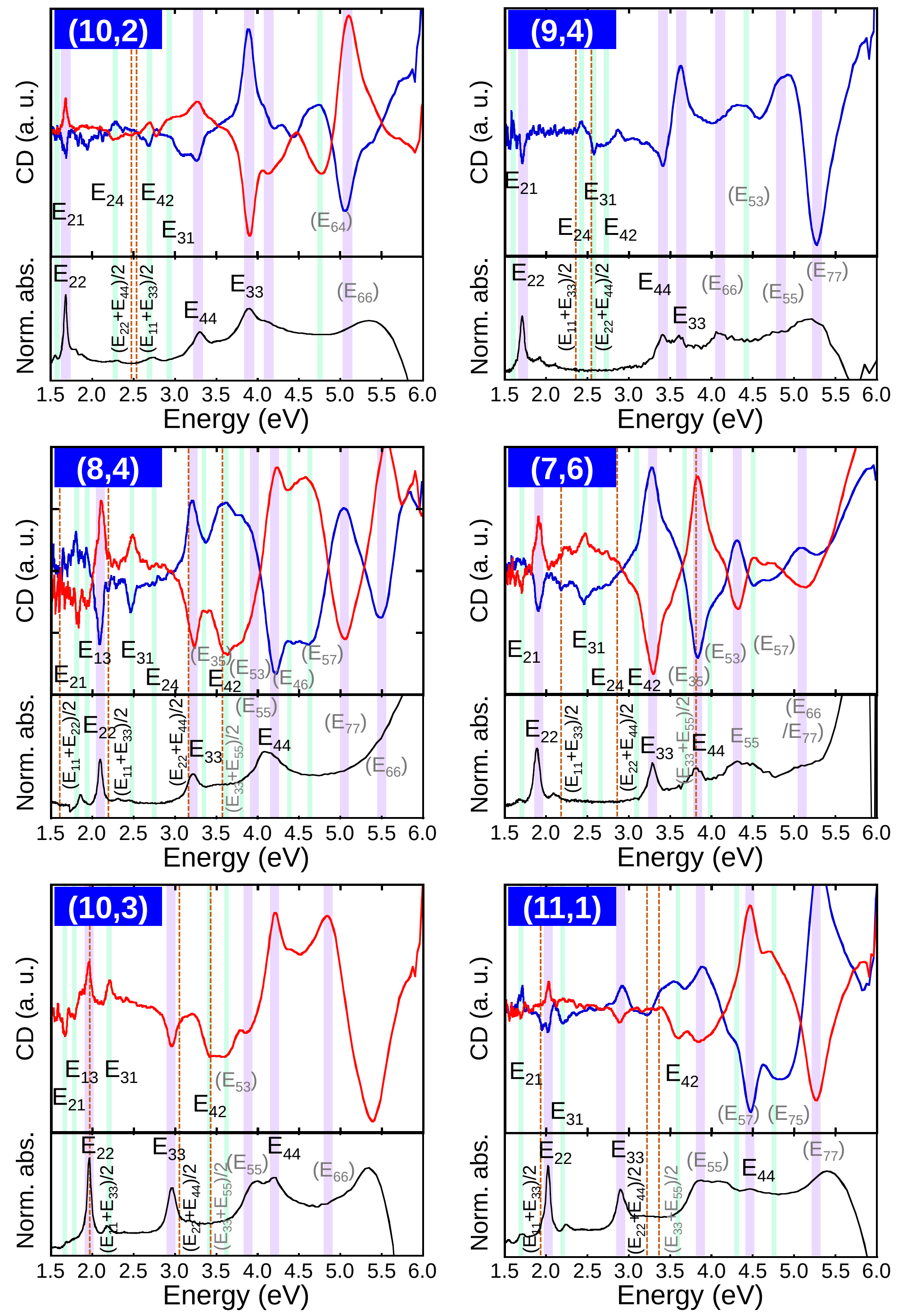

Supplementary Figure 9 (continued). Assignment results of observed optical transitions for 12 (*n*,*m*) SWCNTs.

Vertical dashed lines correspond to the center positions of the pair of E_{ij} transitions. The optical transitions corresponding to Δn = 0 and Δn = ±1 are denoted as purple and green strips, respectively.

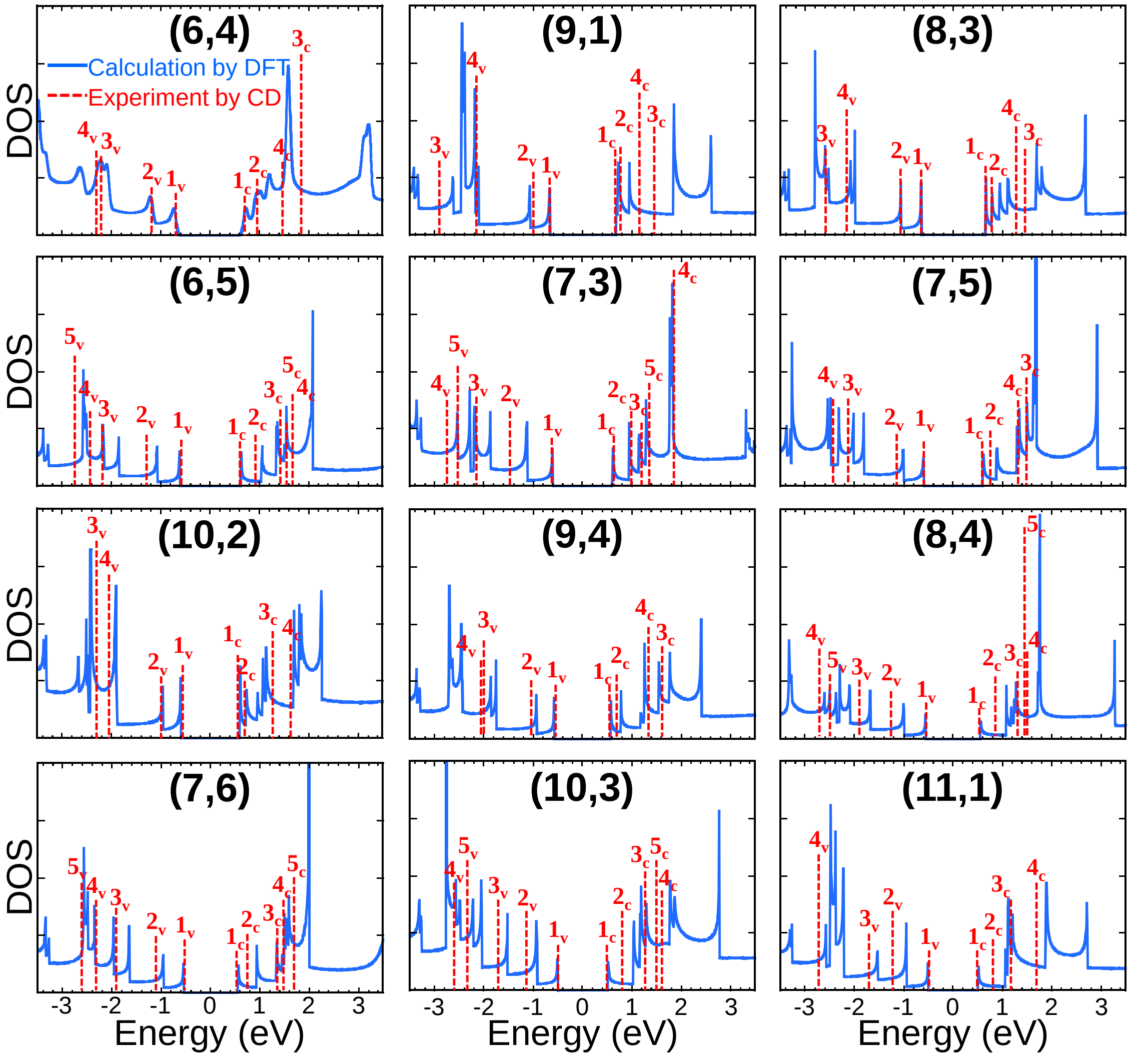

Supplementary Figure 10. Comparison of experimental results based on CD spectra (red dash line) and DOS calculated by LDA[3,4] (blue solid line).

The energy bands (n_c and n_v) in the LDA calculation were shifted away from the Fermi level by +0.15 and –0.15 eV for comparing, respectively.

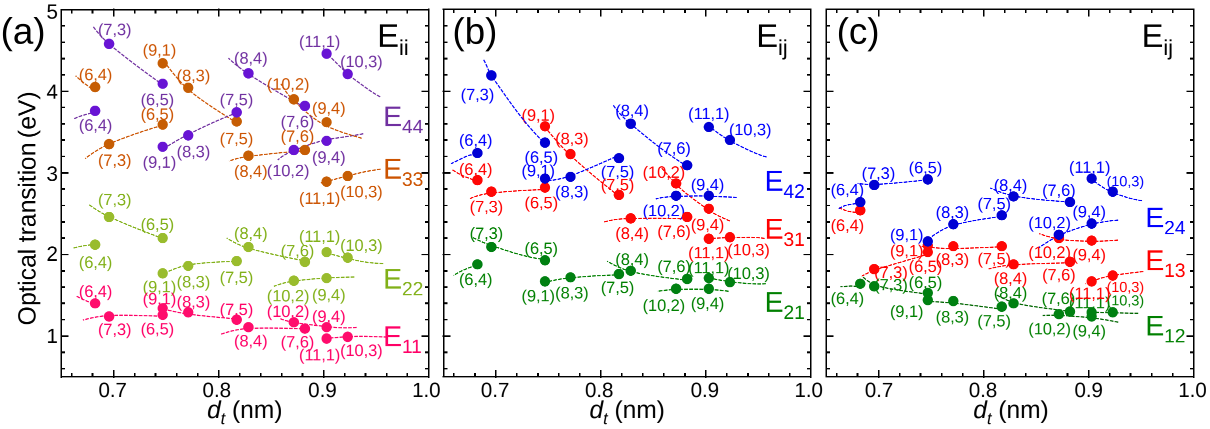

Supplementary Figure 11. Experimentally observed E_{ii} and E_{ij} transitions of the 12 (*n*,*m*) species.

(a-c) Energy plots for experimental E_{ii} and E_{ij} transitions as a function of d_t. The chiralities with the same values of (2*n* + *m*) are connected by a dashed line to guide the eye.

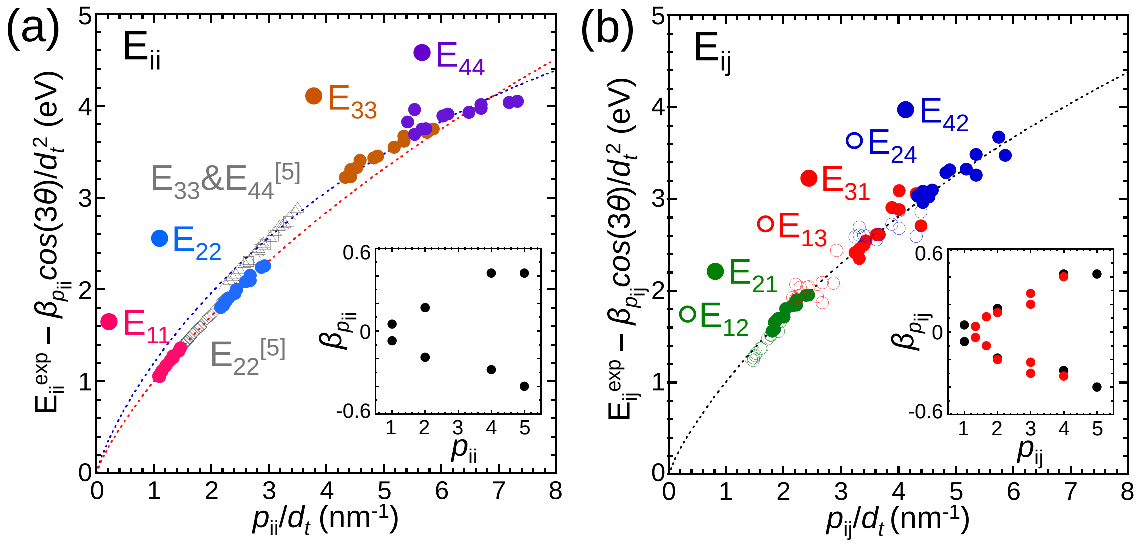

Supplementary Figure 12. Nonlinear scaling behavior of E$_{ii}$ and E$_{ij}$ transitions.

(a) Experimental E$_{ii}$ transition energies after correcting for the chirality dependence ($E_{ii}^{exp} - \beta_{p_{ii}} cos3\theta/d_t^2$), as a function of p_{ii}/d_t. Gray symbols indicate the data from Suppl. Ref. 5 for comparison. Dotted curves indicate E$_{ii}$ energies calculated by the empirical formula, $E_{ii}(p_{ii}, d_t) - \beta_{p_{ii}} cos3\theta/d_t^2 = a\frac{p_{ii}}{d_t}\left[1 + b\log\frac{c}{p_{ii}/d_t}\right]$, given in Suppl. Ref. 5. Parameters a, b, c, and $\beta_{p_{ii}}$ were determined from our experimental results by fitting. Red curve shows result for E$_{11}$ and E$_{22}$ where a = 0.970 eV·nm, b = 0.497, c = 1.154 nm^{-1}. Blue curve shows result for E$_{33}$ and E$_{44}$ transitions, where a = 1.079 eV·nm, b = 0.668, and c = 1.467 nm^{-1}. Inset shows the $\beta_{p_{ii}}$ values for the lower (upper) branch in the family pattern of E$_{ii}$ - d_t plot, –0.07 (0.05), –0.19 (0.17), –0.28 (0.42), –0.40 (0.42) for p_{ii} = 1, 2, 4, 5, respectively. (b) Experimental E$_{ij}$ transition energies after correcting for the chirality dependence ($E_{ij}^{exp} - \beta_{p_{ij}} cos3\theta/d_t^2$), as a function of p_{ij}/d_t, where the p_{ij} values are 1.33, 1.66, 2, 3, 3, and 4 for E$_{12}$, E$_{21}$, E$_{13}$, E$_{31}$, E$_{24}$, E$_{42}$, respectively, from the cutting lines of these transitions. The E$_{ij}$ transitions are fitted by the extended empirical formula, $E_{ij}(p_{ij}, d_t) - \beta_{p_{ij}} cos3\theta/d_t^2 = a\frac{p_{ij}}{d_t}\left[1 + b\log\frac{c}{p_{ij}/d_t}\right]$. Where, fitted parameters are a = 0.932 eV·nm, b = 0.547, and c = 1.412 nm^{-1}. Dotted curves indicate the fitting results for all E$_{ij}$ transitions. Inset: the applied $\beta_{p_{ij}}$ values denoted as red circles for the lower (upper) E$_{ij}$ branches are –0.04 (0.04), –0.10 (0.11), –0.20 (0.14), –0.22 (0.28), –0.30 (0.20), –0.32 (0.40) for E$_{12}$, E$_{21}$, E$_{13}$, E$_{31}$, E$_{24}$, E$_{42}$, respectively. The black circles indicate $\beta_{p_{ii}}$ for comparison.

Supplementary Table.

Supplementary Table 1. Purity (%) of chirality-sorted (*n*,*m*) SWCNTs.

(*n*,*m*)	(6,4)	(9,1)	(8,3)	(6 5)	(7,3)	(7,5)	(10,2)	(9,4)	(8,4)	(7,6)	(10,3)	(11,1)
This work	$97_{(-)}$	$91_{(-)}$	$96_{(-)}$	$98_{(-)}$	$93_{(-)}$	$90_{(+)}$	$90_{(-)}$	$97_{(+)}$	$80_{(+)}$	$94_{(+)}$	$94_{(-)}$	$90_{(+)}$
DGU[6]	88	36	55	83	66	46	40	–	34	48	–	–
DNA wrapping[7]	–	80	70	90	–	90	90	60	90	90	–	–
Gel: repeated overloading[8]	46	–	56	93	89	88	39	46	63	94	69	–
Gel: temp. control[9]	66	–	52	91	–	58	–	–	71	73	–	–

Supplementary Methods

Separation using different SDS concentrations in the first separation.

To obtain single-chiral SWCNT enantiomers that could not be adsorbed in 3% SDS, the SDS concentration was lowered to 2% in the first separation process. The SWCNT dispersion was prepared by sonication and ultracentrifugation in 2% SDS aqueous solution. The SWCNTs dispersion was used for the first separation as described in the manuscript, but the SDS concentration used in the equilibration and the wash of the column was changed to 2%. For the second separation, the SWCNT eluted from the fifth column of the first multicolumn separation was used. The second separation was conducted as described in the manuscript, but the DOC concentrations for the stepwise elution were changed from 0.02 to 0.06%, as shown in Supplementary Figure 3. Consequently, both (+)- and (–)-enantiomers for (9,1) and (8,3) were newly obtained.

Separation of larger diameter enantiomers.

To obtain larger diameter single-chiral SWCNT enantiomers, the unadsorbed SWCNTs fraction obtained from the first multicolumn separation was used as the starting material for the second separation. A net weight of 100 mg of SWCNT powder in 100 ml of 2% SDS solution was sonicated for 6 hours, followed by ultracentrifugation, as described in the manuscript. The prepared SWCNT dispersion was applied to the 4 columns of the multicolumn separation setup. The separation was repeated seven times, and the final unadsorbed fraction was used for the second separation. For the second separation, the same amount of 2%

SDS solution was added to the unadsorbed fraction. The resulting SWCNTs in 0.5% SC + 1.0% SDS solution was used for the second separation conducted in 0.5% SC + 1.0% SDS solution. The DOC concentrations for stepwise elution were changed from 0.07 to 0.165% DOC in 0.5% SC and 1.0% SDS (Supplementary Figure 4). The separation processes were performed at room temperature (20 ± 0.5 °C). Consequently, both (+)- and (–)-enantiomers for (10,2) and (11,1) and (–)-(10,3) enantiomer were newly obtained.

Supplementary References

1. Takase, M. *et al*. Selection-rule breakdown in plasmon-induced electronic excitation of an isolated single-walled carbon nanotube. *Nature Photon.* **7,** 550–554 (2013).

2. Ajiki, H. & Ando, T. Electronic states of carbon nanotubes. *J. Phys. Soc. Jpn* **62,** 1255–1266 (1993).

3. Kato, K., Koretsune, T. & Saito, S. Energetics and electronic properties of twisted single-walled carbon nanotubes. *Phys. Rev. B* **85,** 115448 (2012).

4. Kato, K., Koretsune, T. & Saito, S. Geometries and DOS of geometry optimized single-wall carbon nanotubes, http://www.stat.phys.titech.ac.jp/saito/optCNTs/OptCNT_LDA_DOS_Kato.html (2013).

5. Araujo, P. T. *et al*. Third and fourth optical transitions in semiconducting carbon nanotubes. *Phys. Rev. Lett.* **98,** 067401 (2007).

6. Ghosh, S., Bachilo, S. M. & Weisman, R. B. Advanced sorting of single-walled carbon nanotubes by nonlinear density-gradient ultracentrifugation. *Nature Nanotech.* **5,** 443–450 (2010).

7. Tu, X., Manohar, S., Jagota, A. & Zheng, M. DNA sequence motifs for structure-specific recognition and separation of carbon nanotubes. *Nature* **460,** 250–253 (2009).

8. Liu, H., Nishide, D., Tanaka, T. & Kataura, H. Large-scale single-chirality separation of single-wall carbon nanotubes by simple gel chromatography. *Nature commun.* **2,** 309 (2011).

9. Liu, H., Tanaka, T., Urabe, Y. & Kataura, H. High-efficiency single-chirality separation of carbon nanotubes using temperature-controlled gel chromatography. *Nano Lett.* **13,** 1996–2003 (2013).
